# Establishment of urinary exosome-like vesicles isolation protocol for FHHNC patients and evaluation of different exosomal RNA extraction methods

**DOI:** 10.1186/s12967-018-1651-z

**Published:** 2018-10-11

**Authors:** M. Vall-Palomar, J. Arévalo, G. Ariceta, A. Meseguer

**Affiliations:** 1 Fisiopatologia Renal, Centre d’Investigacions en Bioquímica i Biologia Molecular (CIBBIM), Institut de Recerca Vall d’Hebron (VHIR), Barcelona, Spain; 2 Nefrologia Pediàtrica, Hospital Universitari Vall d’Hebron (HUVH), Barcelona, Spain; 3 Departament de Bioquímica i Biologia Molecular, Unitat de Bioquímica de Medicina, Universitat Autònoma de Barcelona (UAB), Barcelona, Spain; 4 Red de Investigación Renal (REDINREN), Instituto de Salud Carlos III-FEDER, Madrid, Spain

**Keywords:** Urinary exosome-like vesicles, Exosome isolation, Exosomal miRNA, FHHNC

## Abstract

**Background:**

Molecular and cellular pathophysiological events occurring in the majority of rare kidney diseases remain to be elucidated. Familial hypomagnesemia with hypercalciuria and nephrocalcinosis (FHHNC) is a rare autosomal recessive disorder caused by mutations in either *CLDN16* or *CLDN19* genes. This disease is characterized by massive urinary wasting of magnesium and calcium, osmosis deregulation and polyuria. Patients with p.G20D homozygous mutation in *CLDN19* gene exhibit different progression to kidney failure suggesting that beyond the pathogenic mutation itself, other molecular events are favoring disease progression. Due to the fact that biopsy is not clinically indicated in these patients, urinary exosome-like vesicles (uEVs) can be envisioned as a valuable non-invasive source of information of events occurring in the kidney. Exosome research has increased notably to identify novel disease biomarkers but there is no consensus standardized protocols for uEVs isolation in patients with polyuria. For this reason, this work was aimed to evaluate and refine different uEVs isolation methods based on differential centrifugation, the gold standard method.

**Results:**

Characterization by NTA, cryo-TEM and immunoblotting techniques identified the most appropriate protocol to obtain the highest yield and purest uEVs enriched fraction possible from urine control samples and FHHNC patients. Moreover, we tested five different RNA extraction methods and evaluated the miRNA expression pattern by qRT-PCR.

**Conclusions:**

In summary, we have standardized the conditions to proceed with the identification of differentially expressed miRNAs in uEVs of FHHNC patients, or other renal diseases characterized by polyuria.

## Background

Rare kidney diseases include at least 150 different inherited disorders that can affect all the segments in the nephron impairing homoeostatic processes [1]. The high number of rare kidney diseases, their low prevalence and, in many cases, their highly phenotypic variability leads to a poor knowledge of the natural history and mechanisms underlying disease progression.

Familial hypomagnesemia with hypercalciuria and nephrocalcinosis (FHHNC) is a rare autosomal-recessive disorder caused by mutations in either *CLDN16* or *CLDN19* genes [2–4]. The loss-of-function of one of those genes leads to massive urinary wasting of magnesium and calcium causing osmosis deregulation and polyuria. Although two-thirds of the affected patients in Spain are presenting a specific founder mutation (c.59G>A; p.G20D) in *CLDN19* gene, there are important individual differences on the progression to end-stage renal disease (ESRD) [5–10]. This suggests the existence of other non-identified molecular events that contribute to the physiopathology of the disease, promoting ESRD progression. Because kidney biopsy samples, which could allow the study of those intra-renal processes, are not clinically indicated in most patients with FHHNC, blood and urine are the only available samples for the discovery of surrogated markers that might indicate disease progression in those patients.

Exosomes are small (30–150 nm) membrane vesicles of endocytic origin contained into multivesicular bodies and released constitutively through fusion with the plasma membrane [11–13]. Exosomes can act as cell-to-cell communication mediators that trigger intracellular signaling pathways [14–18] and have been pointed out as a promising source of biomarkers, since their cargo (protein, DNA, mRNA and non-coding RNA) represents their cellular origin content [18–21]. The biogenesis and excretion of nanovesicles take place in every segment of the renal nephron [12], thereby, analysis of urinary vesicles should provide information about the pathophysiological state of the entire renal tubule and might be considered a reliable non-invasive source of cellular events information [12, 22]. It is important to remark that due to the absence of exosome specific markers that lead to an uncertain origin of the isolated vesicles (released from multivesicular bodies or other intracellular compartments), urinary exosome-like vesicles (uEVs) are considered as the enriched fraction of nanovesicles having similar size and morphology.

Diverse studies have shown that nanovesicles can be recovered from urine by ultracentrifugation, ultrafiltration or by other techniques [12, 23, 24]; however, these studies focused predominantly on patients with normal urine output. There is no information about the efficiency of those methods in isolating nanovesicles from patients with urinary ionic deregulation and massive urine wasting. In diseases coursing with severe polyuria, such as FHHNC, urinary exosome-enrichment may be lower than in a control sample. Therefore, we decided that differential centrifugation, which allows an unlimited urine volume input, might be the best method to obtain sufficient exosome yields for subsequent analysis. So far, diverse differential centrifugation protocols for urinary exosome isolation have been described in the literature, including: (a) DTT treatment step to release entrapped exosomes from the polymeric Tamm–Horsfall protein (THP) networks, the most abundant protein in urine [25–30]; (b) large extracellular vesicles removal through a 0.22 µm filter to restrict the co-precipitation of larger extracellular vesicles presents in urine [26]; (c) exosome-enriched fraction precipitation speed ranging 100,000–200,000*×g* [26, 28, 29, 31].

To the best of our knowledge, there is no consensus protocol to isolate uEVs of patients with polyuria. Therefore, in the present study we propose an optimized differential centrifugation method for uEVs isolation that would be useful for further identification of markers linked to disease outcome in patients with FHHNC [32].

One of the most appealing features of isolating urinary exosomes is explained by their miRNA cargo. In particular, exosomal miRNAs have become an object of study in many biomedical areas to identify novel and highly promising biomarkers for kidney diseases [20, 33, 34]. In this work, we also evaluated five different RNA extraction methods and selected the one that provided with the best yield and purity for further differential miRNAs expression analysis.

## Methods

In order to standardize a protocol for uEVs isolation in patients with polyuria we evaluated and refined different uEVs isolation methods based on differential centrifugation.

### Urinary exosome-like vesicles isolation

**Fig. 1:**
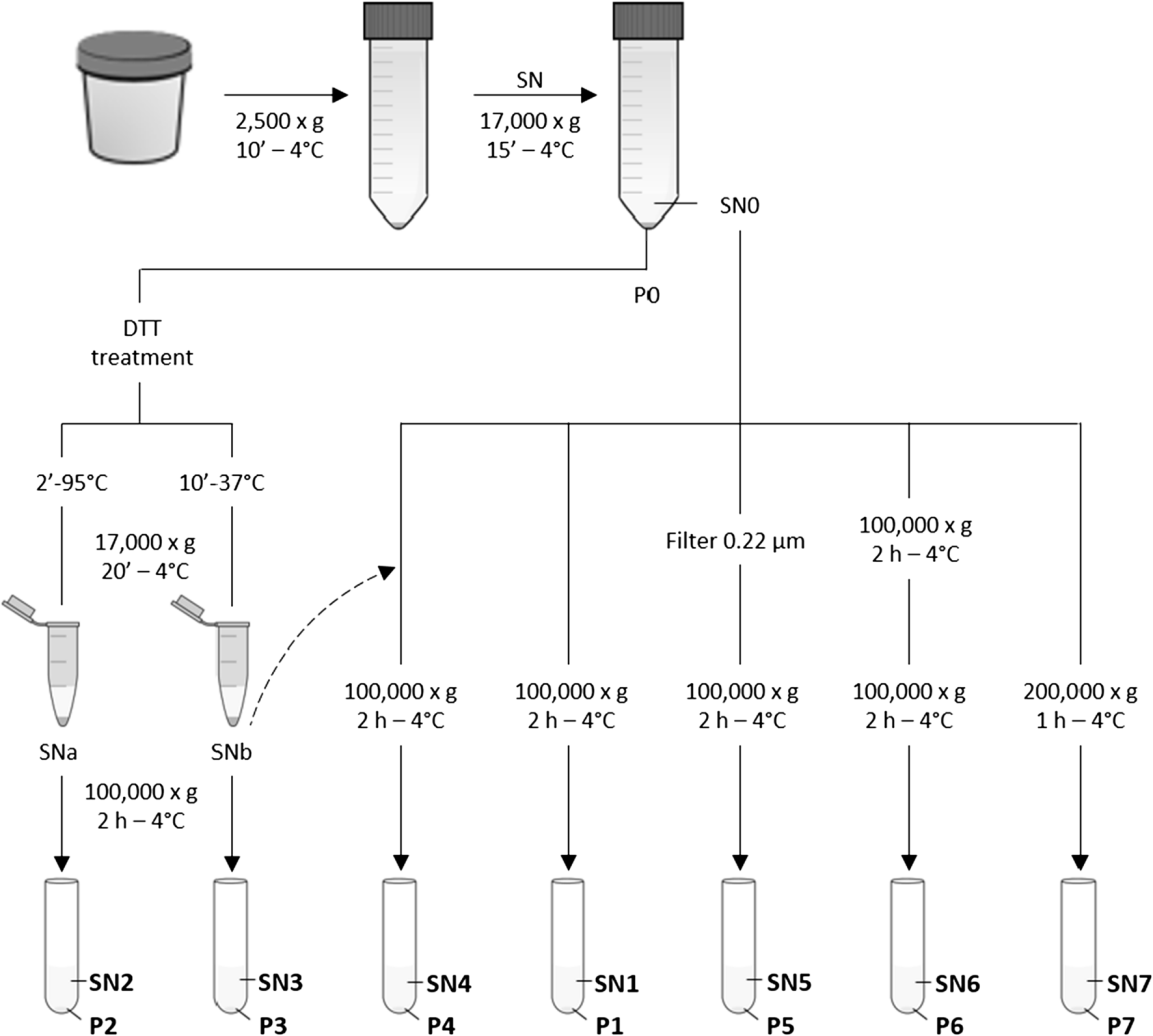
UEVs isolation workflow. Schematic representation of protocols tested to isolate uEVs. Supernatants obtained from different procedures (SN0 to SN7) or treatments (SNa and SNb). uEVs pellets obtained from different procedures (P0–P7)

We performed different approaches (based on differential centrifugation) named P1-P7 (Fig. 1).

Approximately 500 mL of urine from seven healthy human donors (control samples), 4 females and 3 males, were collected in sterile recipients, pooled and maintained at 4 °C for 24 h. These conditions recapitulate those suffered by FHHNC patient’s samples collected at different hospitals of Spain. Protease inhibitor cocktail (PIC) (#S8820 Sigma-Aldrich, Darmstadt, DE) was added to the sample at 1 µL/mL and centrifuged 10 min at 2500×*g* to remove cell debris. Samples were centrifuged 15 min at 17,000×*g* to pull down cell fragments, apoptotic cells and larger vesicles. The supernatant (SN0) was kept on ice while the pellet obtained (P0) was treated with DTT (#A2948 AppliChem, Barcelona, ES) (200 mg/mL) at 95 °C for 2 min or at 37 °C for 10 min and vortexed every 2 min. Both DTT treated pellets were centrifuged at 17,000×*g* for 20 min and supernatants (SNa-SNb) were ultracentrifuged (Sorvall WX90; AH629 rotor) at 100,000×*g* for 2 h, obtaining P2 and P3 respectively.

SN0 was divided into five 100 mL aliquots that were processed by different procedures: (a) SN0 ultracentrifugation at 100,000×*g* for 2 h (P1); (b) combination of SN0 with SNb and ultracentrifugation at 100,000×*g* for 2 h (P4); (c) SN0 filtration through a 0.22 µm-pore followed by ultracentrifugation at 100,000×*g* for 2 h (P5); (d) SN0 double ultracentrifugation for 2 h at 100,000×*g* (P6) and; (e) SN0 ultracentrifugation at 200,000×*g* for 1 h (P7), all them performed at 4 °C. All the uEVs isolated were resuspended in 150 µL of cold and pre-filtered PBS 1× and saved at − 80 °C.

Urine samples from 20 FHHNC patients (8 women and 12 men) were collected at hospitals from different geographical places in Spain and individually processed at Hospital Universitari Vall d’Hebron, as previously described. Clinical and genetic data from these patients is included in the RENALTUBE Spanish Registry (www.renaltube.com) [32].

### Urinary exosome-like vesicles characterization

uEVs obtained following the procedure described above were characterized by three methods: Nanoparticle Tracking Analysis (NTA), Cryogenic Transmission Electron Microscopy (cryo-TEM) and immunoblotting techniques.

#### NTA

NTA was performed using NanoSight NS300 (Malvern Instruments). Videos were recorded and analyzed using NTA software v3.1 (NanoSight Ltd., UK). Background extraction was applied and the automatic setting for minimum track length, blur settings and minimum expected particle size were established. uEVs samples were diluted to optimal concentrations according to the manufacturer’s protocol. Each measure was carried out in triplicate of 60 s recordings at 25 frames per second, generating three replicate histograms that were averaged.

#### Cryo-TEM

Ten microliters of uEVs diluted in PBS 1× were placed on Formvar-Carbon EM grids and frozen in ethane. Samples were analyzed on a Jeol JEM 2011 transmission electron microscope at an accelerating voltage of 200 kV.

#### Immunoblotting

uEVs suspensions were treated with lysis buffer 1:1 (Tris–HCl 50 mM pH 7.5, EDTA 1 mM pH 8, NaCl 150 mM, SDS 0.1%, NP-40 1%, sodium deoxycholate 0.5%, PIC, 1:200, NaF 1 mM, Na_3_VO_4_ 1 mM). After 1 h shaking at 4 °C, samples were sonicated at maximum amplitude for 5 cycles of 5 s and then centrifuged at 13,000×*g* for 15 min at 4 °C. Supernatants were collected and stored at − 20 °C. An equal amount of total uEVs lysates (20 µL) were mixed with Laemmli sample buffer 1:1 (Tris–HCl pH 6.8 0.3 M, SDS 10%, glycerol 5%, β-mercaptoethanol 2%, bromophenol blue 0.01%) and loaded onto 15% acrylamide gel and ran at 100–150 V for 1 h. Proteins were transferred to a PVDF 0.2 µm membrane (Immobilon-P^SQ^) (#ISEQ00010 Millipore, Darmstadt, DE) at 100 V for 1 h. Membranes were blocked using 5% non-fat dry milk diluted in PBS-T (PBS 1×, Tween-20 0.1%) and tested against specific primary antibodies: TSG101 (#Ab83 Abcam, Cambridge, UK) 1:500 and Alix (#Ab76608 Abcam, Cambridge, UK) 1:500. Secondary antibodies were rabbit anti-mouse polyclonal and goat anti-rabbit polyclonal, respectively (#P0260, #P0448 Dako, California, EUA) 1:5000. All the antibodies were diluted in blocking solution.

Immunoblots were visualized using chemiluminescence reagent (#WBLUF0500 Millipore, Darmstadt, DE) and Odyssey Fc Imaging System (Li-Cor, Lincoln, NE).

### RNA extraction and miRNA expression pattern

#### uEVs RNA extraction and quantification

For total RNA extraction, 5 different methods were tested: miRNeasy Mini kit (#217004 Qiagen), miRCURY RNA Isolation kit (#300110 Exiqon, Vedbaek, DK), Allprep DNA/RNA/miRNA Universal kit (#80224 Qiagen), TRIzol LS (#10296010 Life Technologies) and TRIzol (#15596026 Life Technologies). Each RNA extraction was performed from 100 µL of FHHNC patient’s uEVs. Purified RNAs were eluted in RNAse-free water and stored at − 80 °C. RNA concentrations were measured by capillary electrophoresis (Agilent 2100 Bioanalyzer-Picochip).

#### miRNA profiling

Four nanograms of total RNAs were retrotranscribed according to Universal cDNA Synthesis Kit (#203301 Exiqon, Vedbaek, DK) protocol. cDNAs were diluted at 1:40 and combined with SYBR Green master mix following the miRCURY LNA Universal microRNA PCR (#203403 Exiqon, Vedbaek, DK) instructions. qPCR was performed in an ABI7500 Real-Time cycler (Applied Biosystems). Primers used were designed to detect: miR-200c-3p, miR-103a-3p, miR-let7b-5p, miR-99a-5p and miR-10b-5p. Unisp6 was added as an exogenous control, since they have been found in uEVs [27].

## Results

### Optimization of urinary exosome-like vesicles isolation protocol

Because of the lack of methodological consensus and the imperative need to standardize the uEVs isolation protocol, we tested several paths based on differential centrifugation steps and sample treatments to determine the procedure that provides the purest and highest uEVs yield (Fig. 1). Morphology, size and quantity of isolated uEVs from a control urine sample pool were evaluated by cryo-TEM and NTA.

**Fig. 2:**
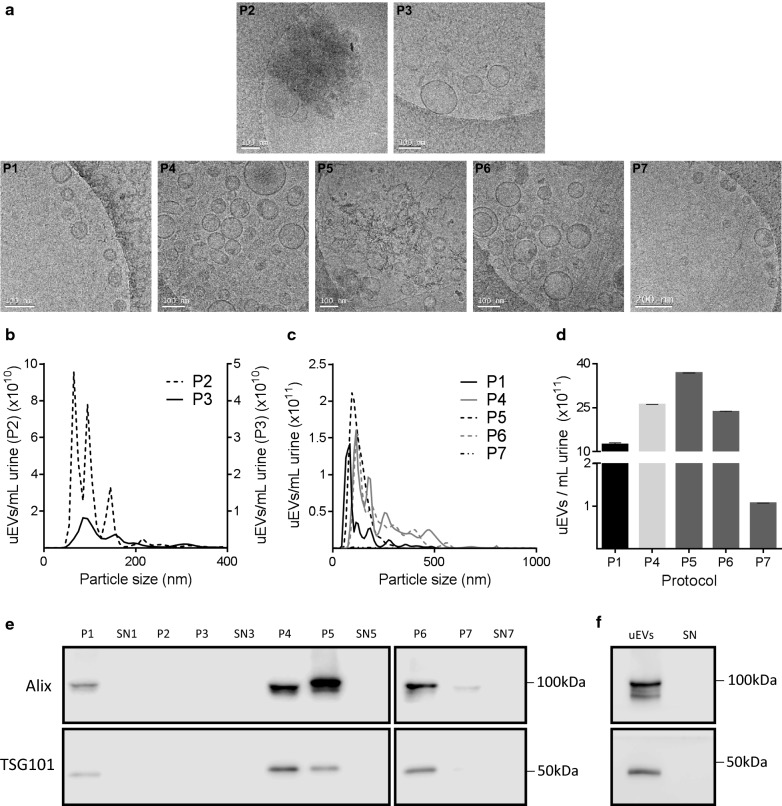
uEVs characterization. **a** Representative cryo-TEM images of all the uEVs pellets obtained from different isolation procedures. Scale bar is 100 nm except in P7 which is 200 nm. **b** NTA results of the uEVs recovered after DTT treatment. P2 showed higher peaks than P3 that might correspond to impurities observed by cryo-TEM. **c** NTA profiles of the uEVs pellets obtained from the five different uEVs isolation protocols. In all cases, the mode size falls within the expected size range. Even though the highest peak is observed in P5, the associated cryo-TEM image suggests that the peak might correspond to impurities. **d** Bar graph showing uEVs concentration by NTA. Because of P5 impurities, NTA quantification may not be accurate. P4 and P6 contained the highest amount of uEVs. Data is expressed as the mean ± SD. **e** Equal volumes of uEVs (20 µL) were loaded for immunoblotting against Alix and TSG101. The strongest signal was obtained in P4, followed by P6. Even though a strong band of Alix is observed in P5, this is slightly above the expected weight. **f** Immunoblotting of uEVs isolated from 300 mL of FHHNC urine, using procedure P4, showed a strong intensity of bands corresponding to Alix and TSG101, indicating uEVs presence and its absence in the supernatant

We first aimed to observe which temperature and time of the DTT treatments tested improved the yield and purity of uEVs. As shown in Fig. 1, the former 17,000×*g* pellet (P0) was treated with DTT either at 95 °C for 2 min or at 37 °C for 10 min and centrifuged at 17,000×*g* for 20 min. The uEVs-containing supernatants (SNa and SNb) were then ultracentrifuged at 100,000×*g* for 2 h, giving as a result pellets P2 and P3. Cryo-TEM images clearly demonstrated that P3 was superior on purity when compared with P2, indicating that DTT treatment for 10 min at 37 °C (P3) was more efficient depolymerizing the THP network than 2 min at 95 °C (P2) treatment (Fig. 2a above). By using NTA we quantified more uEVs in P2 than in P3 (Fig. 2b) likely due to the presence of impurities (THP complexes) contained in P2. Western blot assays for Alix and TSG101 proteins gave negative results for P2 and P3, in accordance with the low yield of exosomes released from the THP network (Fig. 2e).

We next analyzed whether recovering THP network entrapped uEVs might improve the final uEVs yield. For this purpose, we mixed SN0 with SNb (Fig. 1) and proceed with the ultracentrifugation at 100,000×*g* for 2 h, obtaining P4. Cryo-TEM images showed that P4 contains highly purified uEVs (Fig. 2a below) that are in higher quantities than those observed in P1 and P3, separately, upon NTA quantification (Fig. 2b, c). To validate uEVs yield by another method and to verify the presence of exosome-enriched proteins in the sample, we performed immunoblots against two exosomal associated proteins (Alix and TSG101), showing higher expression of these proteins in P4 than in P1 (Fig. 2e). In conclusion, we could say that mixing uEVs released from the DTT treated pellet with SN0 increases the number of uEVs without compromising its purity.

NTA profiles of uEVs obtained from the different isolation procedures revealed that mode size falls within the expected size range. NTA quantification confirms that P5, obtained after one ultracentrifugation of filtered SN0 at 100,000×*g* for 2 h, exhibits the highest yield, followed by P4 (described above), P6 (SN0 at 100,000×*g* for 2 h twice), P1 (SN0 at 100,000×*g* for 2 h once) and P7 (SN0 at 200,000×*g* for 1 h once) (Fig. 2c, d). These results are in accordance with the visual inspection of uEVs by cryo-TEM, except for P5 which seemed to drag some unknown material by the filtering process (Fig. 2a below). Western blot assays did also agree with previous NTA quantification (Fig. 2e). Supernatants SN1, SN3, SN5 and SN7, resulting from uEVs precipitation steps, were included as controls to demonstrate that no exosome particles remain in those supernatants.

Overall, data obtained in this study indicate that: (i) the protocol used in P5 produces the best yield but the cryo-TEM images show impurities in the sample that makes it unacceptable for further differential expression analyses of miRNAs; (ii) the protocol used in P7 is not producing enough exosomes for our purposes; (iii) the procedure used in P4 is the one that shows the best yield and purity followed by the one used in P6. In conclusion, ultracentrifugation at 100,000×*g* for 2 h of mixed SN0 and SNb was selected as the preferred protocol for uEVs isolation and was the one validated and used in patient’s urine samples.

In order to validate the proposed methodology in FHHNC patients, whose urine is estimated to be diluted at least three times in relation to control urine, we performed the P4 procedure in 300 mL of patients’ urine and observed that the selected protocol is suitable for successful uEVs isolation from diluted urines (Fig. 2f).

### Evaluation of different RNA extraction methods and miRNA expression pattern

**Fig. 3:**
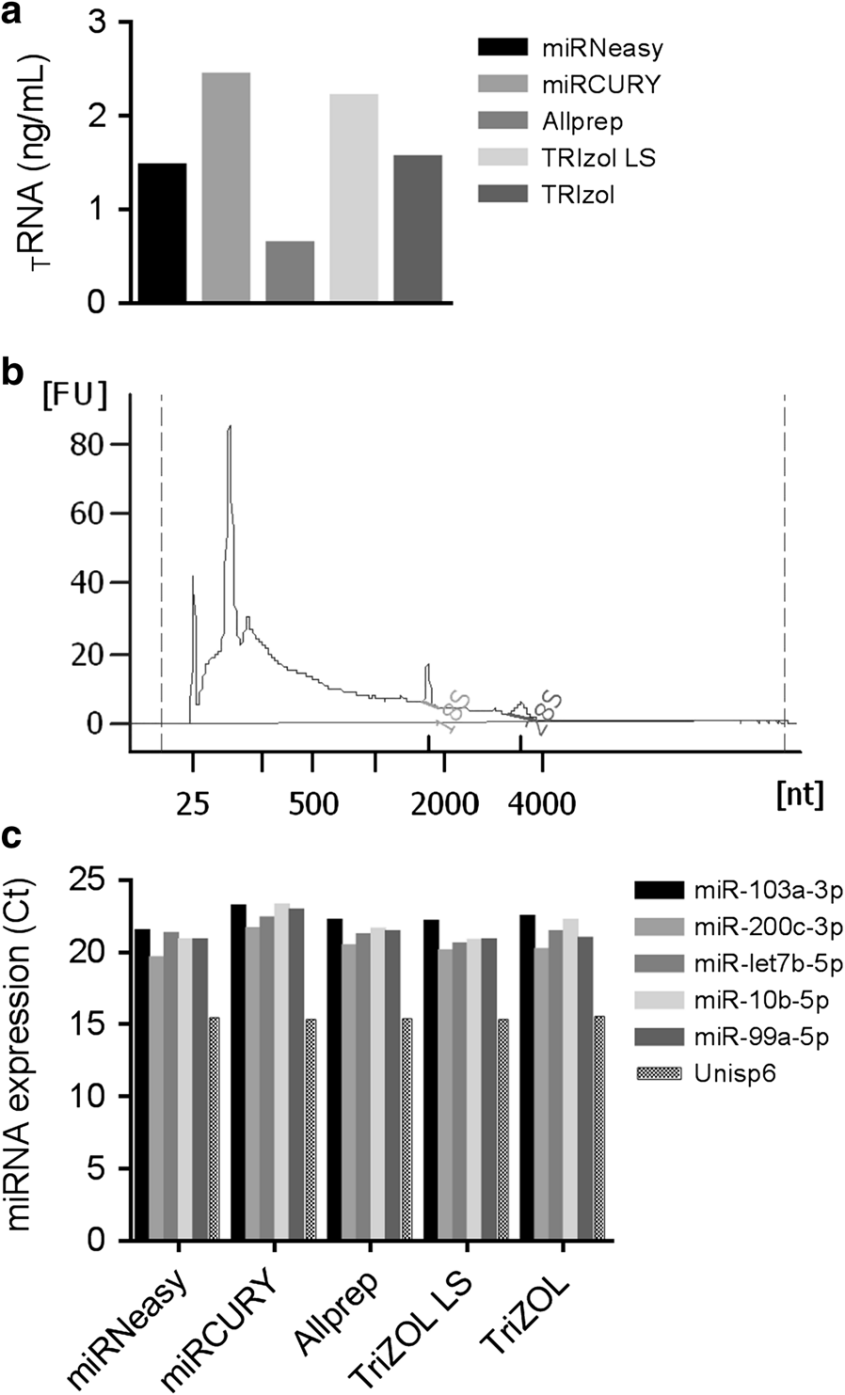
RNA quantification. **a** Bar graph of total RNA quantified from FHHNC uEVs by Bioanalyzer—Picochip using the five different extraction methods. MirCURY kit followed by TRIzol LS were the most efficient methods. **b** A representative electropherogram shows that uEVs contain small RNA, including microRNAs (10–40 nt). As expected, small amounts of rRNA were detected. **c** miRNA profiling by RT-qPCR of RNA extracted from FHHNC uEVs. miRNA expression pattern is consistent independently of the RNA extraction method

A systematic comparison of five different commercial RNA extraction methods (mentioned above) was performed with uEVs isolated from a FHHNC patient. Total RNA, including miRNAs, were extracted from 100 µL of uEVs in each procedure, being miRCURY and TRIzol LS the most efficient methods, according to the Agilent 2100 Bioanalyzer-Picochip quantification analyses (Fig. 3a). The miRCURY-RNA profile obtained with Bioanalyzer-Picochip is shown in Fig. 3b. To evaluate if the RNA extraction method could affect the miRNA profile of the samples, we analyzed five different miRNAs by qRT-PCR (miR-103a-3p, miR-200c-3p, miR-let7b, miR-10b-5p and miR-99a-5p). Unisp6 was added as an exogenous control. Results obtained in Fig. 3c demonstrated that miRNA pattern is similar in all samples, and therefore that the RNA extraction method does not have any significant effect on miRNA profiling. Unisp6 levels were exactly the same for each procedure, validating the assay.

## Discussion

Urine sample analysis is irreplaceable as a non-invasive method for disease diagnosis and follow-up, particularly for kidney diseases. However, non-degraded protein and RNA may be only found in uEVs. Unlike tissue biopsy, an invasive and expensive procedure that allows only a partial sampling of an organ, uEVs provide a full representation of the entire urinary system and their study is attractive in the field of biomarker discovery in kidney diseases. RNA and proteins confined into uEVs can be used as a reliable and non-invasive source of information of renal cellular and molecular related-events.

There are a growing number of commercial kits for uEVs isolation but the results often differ from those obtained using traditional isolation techniques. In diseases such as FHHNC, coursing with polyuria, the starting quantity of urine required to obtain a good yield of uEVs might be higher than in healthy individuals and the currently available exosome isolation kits are not suitable for our purposes. Therefore, for the aim of this pilot study, which is in its discovery phase, we propose the use of ultracentrifugation techniques, which are the gold standard method for uEVs isolation and allow an unlimited urine volume input to obtain enough uEVs in highly diluted urine samples.

Diverse protocols based on differential centrifugation have been described in several reports [27, 28, 35–39]. However, there is no a consensus protocol that would provide information on the amount of starting urine volume, nor a detailed and thorough procedure that could give reliable information on uEVs yield and purity. Because we want to use these uEVs for further comparative miRNA profiling of patients suffering from FHHNC, the aim of this study was to standardize a differential centrifugation protocol for uEVs isolation from diluted samples. We validated every step of the ultracentrifugation process by cryo-TEM, NTA and immunoblotting assays, to finally establish a protocol that would be suitable for the accomplishment of our goals and also to select the best RNA extraction method for further miRNA profiling.

The first issue that we faced was sample handling standardization and storage. In rare diseases, such as FHNNC, the number of patients is very low and they are distributed in different places of the geography which requires sample transportation before uEVs isolation. Urine, which is a complex fluid, should not be frozen before removing cells and cellular debris that can be lysed releasing their content in an undesired manner. For this reason, and although previous studies recommended freezing samples at − 80 °C to loss the minimum urinary exosome-associated proteins after urine collection [40], we maintained control sample at 4 °C for 24 h, in order to mimic the time-lag and temperature of transportation, and proceed to uEVs isolation afterward. Our results indicated that this storage procedure maintained uEVs structure in perfect conditions for further analysis, as demonstrated by cryo-TEM.

Regarding DTT treatment, there are different recommendations in the literature in terms of time and temperature [25–30]. Our results showed that incubation at 37 °C for 10 min (P3) is the condition that produced the purest uEVs, as well as, an acceptable yield (Fig. 2a above and Fig. 2b).

Different speed and ultracentrifugation cycles of SN0 showed that 100,000×*g* for 2 h (P1) is more efficient than 200,000×*g* for 1 h (P7). Moreover, uEVs yield was improved with the addition of uEVs released from THP networks (SNb) to SN0 (P4). Another step analyzed was the filtering of SN0 through a 0.22 µm filter [19, 41–43]. Unexpectedly, cryo-TEM analysis indicated that filtered uEVs contained many impurities (Fig. 2a below), likely released by the filter or from disrupted uEVs as a consequence of pressure, producing an over-quantification by NTA (Fig. 2c, d). The efficiency of ultracentrifugation in uEVs isolation was confirmed by immunoblotting since neither Alix nor TSG101 were detected in supernatants (Fig. 2e). In accordance with our results, we selected P4 as the best protocol to process diluted urine from FHHNC patients which is, on average, three times more diluted than controls, adapting the protocol to 300 mL of starting urine instead of 100 mL. The presence of exosomal-enriched markers (Fig. 2f), indicated that the volume sample input was enough to obtain a sufficient yield of uEVs.

An accurate quantification of RNA extracted from uEVs, which mainly includes miRNA, represents an important bottleneck for comparative expression analyses. Since there are no robust endogenous controls for data normalization of miRNA from uEVs, quantification should be as accurate as possible. Our next concern was then to obtain and quantify, in a reliable manner, the RNA yield from the FHHNC uEVs. Five RNA extraction methods were compared in different aliquots of the same uEVs sample.

Quantification of the RNA obtained by each method was based on spectrophotometry (Nanodrop ND-1000), capillary electrophoresis (Bioanalyzer—Nanochip and Picochip) and fluorescence (Ribogreen) (data not shown). In our experience, the Bioanalyzer Picochip, with a detection range from 50 to 5000 pg/µL, was the most precise, consistent and robust method for RNA measurement. Therefore, it was used as the reference for other RNA quantification methods and selected for further uses. We observed that Nanodrop and Ribogreen over- and under-estimated RNA quantifications, respectively, and Bioanalyzer Nanochip, with a detection range from 5 to 500 ng/µL, provided non-consistent results. Subsequent qRT-PCR, showing similar expression levels of each miRNA among all samples demonstrated the accuracy of the Bioanalyzer Picochip method. Albeit the similar miRNA expression pattern obtained from the five different RNA extraction methods (Fig. 3c), we selected miRCURY kit since exhibited the highest RNA yield.

This work has provided with a comprehensive protocol for reliable uEVs isolation, both from healthy individuals and polyuric patients suffering from kidney disease. It covers the conditions for sample pre-processing, uEVs isolation, characterization and quantification, as well as, the conditions for RNA isolation and quantification. This protocol shall be useful for differential high-throughput miRNA expression assays between healthy individuals and FHHNC patients that might eventually result in the identification of novel biomarkers of disease progression and/or therapeutic targets.

## Conclusions

We established the best protocol for uEVs isolation in control samples based on ultracentrifugation at 100,000×*g* for 2 h of mixed 17,000×*g* supernatant (SN0) and its pellet treated with DTT for 10 min at 37 °C (SNb). This selected protocol was also suitable to threefold diluted urine from patients coursing with polyuria such as FHHNC patients. Moreover we demonstrated that miRNA pattern is independent of the RNA extraction method being miRCURY kit and TRIzol LS, with which we obtained the highest RNA yield.
